# An illustration of how responsive feedback in a social marketing tobacco control intervention in Ghana enabled managers to make decisions that increased intervention effectiveness

**DOI:** 10.12688/gatesopenres.13062.1

**Published:** 2019-09-09

**Authors:** Sohail Agha, Jean Paullin

**Affiliations:** 1 Global Development, The Bill & Melinda Gates Foundation, Seattle, Washington, 98040, USA; 2 Global Policy and Advocacy, The Bill & Melinda Gates Foundation, Seattle, Washington, 98040, USA

**Keywords:** Implementation science, responsive feedback, feedback loops, adaptive implementation, theory of change, monitoring and evaluation

## Abstract

This report illustrates how a feedback loop, set up to provide data and insights to a donor and designers/implementers of a social marketing tobacco prevention intervention in Ghana, helped adapt the original design of the intervention to one that was more suited to the social and media contexts of Ghana. The designers/implementers had previously, successfully implemented a tobacco control intervention with adolescents in Botswana. This experience had informed the initial intervention design in Ghana. As the feedback generated by evaluators started demonstrating just how different the Ghanaian social and media contexts were from the Botswanan one, implementers started making changes to their selection of channels, resulting in a design which was quite different from the original one. The close involvement of the donor in this process enabled implementers to make rapid changes to the design of the intervention. This illustration adds to a small but growing literature establishing the importance of feedback loops to improve the design and implementation of development interventions.

## Disclaimer

The views expressed in this article are those of the authors. Publication in Gates Open Research does not imply endorsement by the Bill & Melinda Gates Foundation.

## Introduction

 “Why do some behavioral interventions succeed and others fail?” is a question at the forefront of every implementer’s mind. Are there systematic processes that, if followed during the design and implementation of a behavioral intervention, increase the likelihood of the intervention’s success? Specifically, can timely learning or responsive feedback during the implementation of an intervention help increase the likelihood of a project’s success?

The answer to this question occupies the attention of “the Curve” community of practice (CoP) supported by the Bill & Melinda Gates Foundation ( Viswanath *et al.,* 2019). This CoP provides a platform to 20 leading implementation and research organizations and 5 donors that, as a group, have focused on this question since 2018. The Curve provides participating organizations the opportunity to think deeply about this question, to learn from each other’s experience and to develop illustrative examples of responsive feedback, with the objective of providing examples which may be of broader interest within the development field.

Here, we illustrate how feedback can help by providing an example of the process of learning during the implementation of a behavioral intervention in Ghana. In 2016, the tobacco control team within the Global Policy and Advocacy (GPA) team at the Bill & Melinda Gates Foundation decided to fund an impact evaluation of a social marketing campaign to prevent tobacco use in Ghana. The tobacco team felt that an objective impact assessment would not only help guide the design and measurement of future social marketing campaigns, but could also contribute to an investment case for prevention-related communication. A preventive approach for tobacco control is extremely important in Africa, where the prevalence of tobacco use is still relatively low and the tobacco industry sees a prime opportunity for market growth. The impact evaluation was designed to respond to several critical questions: which messages are most effective; which media are most effective; how cost-effective is the campaign; and, perhaps most importantly, what is the magnitude of the campaign’s impact.

The implementing organization, *Good Business* (GB), had previously designed social marketing interventions in Botswana and Uganda. These interventions had been implemented with the aim of changing knowledge, attitudes, and perceptions around tobacco use. GB had hired researchers to measure the effects of its intervention among adolescents who were exposed to project activities through indicators such as the number of girls who spontaneously listed smoking as something they were opposed to and the number of girls who thought smoking made them more attractive. This ability to track behavior among teens exposed to the intervention had enabled GB to feel confident about continuing with the implementation of planned activities. While results from the campaigns were generally positive, the foundation’s tobacco control team wanted independent verification that the approach worked, and that positive changes in attitudes and norms related to smoking could be attributed to the campaign. In other words, they wanted a comparison of the effects of the campaign with what would have happened in its absence. The tobacco control team felt that a population-based impact evaluation would add to the evidence base and help them build an investment case for using social marketing to prevent teen tobacco use in sub-Saharan Africa.

The *Tulane University School of Public Health and Tropical Medicine* (Tulane) was selected to conduct the evaluation using a quasi-experimental, panel design. The evaluation relied on population-based household surveys conducted with randomly selected 13–17 year-old girls in project and non-project areas in Ghana. The full methodology and results of the evaluation are detailed in Hutchinson *et al.,* 2019a. These surveys were conducted before activities were initiated and at the end of the project. A unique, additional feature of the evaluation was a feedback loop comprised of multiple rounds of mobile phone surveys designed to measure exposure and message comprehension among the project’s target audience during the project’s 20-month implementation period. The level of exposure to a social marketing intervention is considered a key determinant of its anticipated success ( Hornik, 2002). Indeed, some experts will not consider assessing the population level impact of an intervention that reaches less than 20% of its target audience.

## Intervention description

**Figure 1.**
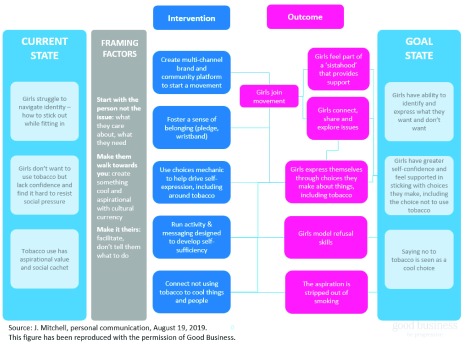
Theory of change for SKY girls tobacco control.

The SKY campaign built upon GB’s experience of implementing a social marketing teen tobacco prevention intervention in Botswana. The Botswana campaign had shown how important a determinant peer influences were on teen tobacco use ( Persons, 2017). Figure 1 shows the Theory of Change (ToC) of the SKY Girls tobacco control intervention. Based on the Botswana experience, the Ghanaian ToC emphasized the importance of a multi-channel brand and community platform to motivate teenage girls to join a movement that fostered a sense of belonging. By connecting to other girls and by expressing themselves through positive choices, the ToC expected adolescent girls to gain self-confidence and a sense of identity that would enable them to reject tobacco use.

In Ghana, formative research conducted by the implementer to design the campaign showed that teen girls were usually aware of the health harms of tobacco and were reluctant to smoke, but the paramount need for social inclusion among fellow teenagers often dictated their decision-making. Teen girls in Ghana lacked the confidence to resist the social pressure of smoking. The choice to smoke had become part of a teenage girl’s struggle with identity formation.

The SKY social marketing campaign was designed to unite girls behind the idea of ‘being true to themselves’, to create an environment that enabled girls to express their true feelings, including their desire not to smoke, and to feel supported by a community of peers. In partnership with the Ghanaian advertising agency *Now Available Africa*, GB launched a platform focused on girls’ empowerment and resilience. Using content around passion points such as music and fashion to draw girls in, the campaign weaved in messaging about choosing not to smoke as the smart, ambitious, aspirational choice.

To reinforce the message, the campaign was executed across multiple media channels, including free magazines, radio programming, free original movies, social media, and live events in Accra. The local agency worked closely with teens to develop content owned by the audience and in direct response to their interests. As part of this campaign, Ghanaian teens were featured throughout the media channels – in the magazine, in live discussions on radio, and on social media.

## Project management

We focus our discussion on the interaction between the implementers and the evaluators, on the type of feedback provided and how the feedback provided enabled implementers to adapt the design of the intervention to the media and cultural context of Ghana.

A primary program officer (JP) from the tobacco control team of the Bill & Melinda Gates Foundation managed both the implementation (primed by GB) and the evaluation (primed by Tulane) grants with support on the evaluation component from a colleague in the foundation’s Global Delivery team. The primary program officer believed that evaluators should maintain their independence but should provide insights and learnings to the implementer on a regular basis to optimize the use of data in management decision-making. The evaluation grant was designed to work in tandem with the implementation of the project: research timelines were aligned with implementation timelines, indicators were linked to the project’s theory of change (ToC).

With this management model in mind, in December 2016, the primary program officer organized an in-person, project kickoff meeting attended by the UK-based implementer, the US-based evaluator and the Seattle-based donor. This provided an opportunity to discuss project objectives and evaluation approaches. Following the kickoff, a cadence of monthly Skype calls was put in place which included the primary program officer, her colleague from Global Delivery (SA), the implementer and evaluator. These meetings occurred consistently over the 20-month duration of project implementation.

From the outset, evaluators focused on learning about the project design, its underlying assumptions and the activities planned. Understanding the project’s ToC and learning about the outcomes expected from the planned activities had immediate implications for the design of survey instruments used for assessing exposure to project activities and measuring the impact of the campaign on outcomes.

As part of their contribution to this interaction, GB reviewed and commented on survey instruments and provided input on measurement indicators they considered most meaningful from a programmatic perspective. For example, given their previous experience with the challenges of retaining very young adolescents’ attention during an interview, implementers insisted on shortening the length of the household survey questionnaire. Once the baseline survey had been completed, Tulane shared the findings with GB for their interpretation and programmatic decision-making.

## Impact evaluation findings

The findings showed that exposure to the SKY campaign was high in Accra. Of girls who lived and attended school in Accra, 84% were exposed to at least one of the SKY channels and 38% were exposed to four or more channels. Of the girls exposed to SKY, 58% said SKY was important to them, with 39% citing the free magazine as the best way to connect to SKY.

The data suggests that the campaign was able to reduce the pressure girls felt to smoke cigarettes and increase teen girls’ confidence in making decisions for themselves. About half of girls exposed to the campaign felt that SKY helped them to reject *shisha* and cigarettes, think about their future and personal goals and feel confident to make decisions for themselves ( Hutchinson *et al.,* 2019b). Four out-of-ten girls exposed to SKY felt that it helped them connect with other girls in their peer group – a part of GB’s goal of building a community of teens that supports girls in rejecting the things they didn’t want.

In the multivariate analysis, the intervention was associated with an 11 percentage point decrease in the perceived pressure to smoke cigarettes, a 10 percentage point increase in the likelihood of conversations with friends about smoking, and similar magnitude of increases in girls’ perceived ability to make choices and in their disagreement with the justification for *shisha* use.

## Feedback loops

In Botswana, GB had used a multi-channel strategy which used a radio show hosted by teen girls, television, the SKY Girls magazine and outdoor events. Social media was a critical component of the Botswana social marketing teen tobacco control campaign. The SKY Girls Facebook page was the hub of the community, allowing girls the freedom to express what they liked (“ **s**ure **k**a **y**one”/SKY) and what they did not (“shapo ka yone”), to share their views and to be supported by other girls. The SKY Girls Facebook page was the third most popular page for teen girls in Botswana and was central to the social marketing campaign’s strategy of amplifying the message.

In contrast with Botswana, the Ghana baseline household survey, conducted by the evaluators prior to the start of the campaign, showed that radio listenership was extremely low among Ghanaian teenage girls. A large proportion of Ghanaian girls were not available online. The use of Facebook, which had been central to the campaign in Botswana, was relatively low in Ghana. Adding to the complexity, television could not be used in Ghana because of the risk of campaign messages contaminating the comparison group. Taken together, this meant that a substantially different channel strategy would have to be adopted for the social marketing campaign in Ghana with no reliance on mass media and reduced reliance on social media.

Several other factors made the Ghanaian context different. Adolescent girls’ mobility was much more restricted in Ghana than in Botswana. To the implementers, this highlighted the importance of schools as a place to reach adolescent girls. School-based activities, which were limited in Botswana because they were considered “uncool”, and were not part of the original plan in Ghana, became vital for the social marketing campaign in Ghana. School-based activities helped build awareness of the brand quickly without the need to advertise on television. The local advertising agency used, *Now Available Africa*, recommended schools as a great means of reaching girls and ensuring a depth of engagement and being more cost-effective. They also emphasized that school activations were not considered “uncool” in Ghana.

It took several months after the baseline household survey for the mobile phone surveys to be implemented. These phone surveys began providing data on campaign exposure. GB were surprised by the initial low level of exposure. This also meant that the number of teens captured in the phone survey who were exposed to SKY was small – limiting an analysis of the profile of Ghanaian girls who were exposed to the campaign - an analysis that is extremely important for marketers to ascertain whether a campaign is reaching those it plans to reach.

To overcome this limitation and provide insights on girls who were exposed to the campaign and to study message comprehension and message effectiveness, the evaluators decided to analyze the SKY Girls database. This was a database on teens exposed to project activities, maintained by GB and *Now Available Africa*. Analysis of this database was not originally part of Tulane’s scope of work. The analysis showed that Facebook was less effective in delivering messages than the SKY Girls magazine. Earlier analysis of the baseline household survey had shown that a small proportion of adolescent Ghanaian girls used Facebook. Based on both insights, the implementers chose to reduce use of Facebook as a platform for messaging about the harms of tobacco, and instead used it as a medium to engage adolescents on how they made decisions on important issues. Thus, both the channel and messaging strategy changed based on insights from multiple sources of data. GB felt that they were able to adapt their content strategy to match the way in which adolescent girls were using different communication channels.

As the approach taken in Ghana was considerably different from the one in Botswana, it was important for implementers to feel confident that the programmatic strategy they were choosing – a strategy that was very different from what they had expected to use – led to increased exposure to SKY among teenagers.

Subsequent rounds of mobile phone surveys showed steadily increasing levels of exposure over time. Surveys showed that the channels being used in Ghana were working and that there was a steady increase in exposure to the campaign. Analysis showing that adolescents really liked the project brand helped increase the implementer’s confidence in the new approach.

The intervention evolved considerably, as results from multiple rounds of mobile phone surveys came in. Rather than relying on Facebook and interactive radio shows, schools became the catchment area for the intervention. The implementer had expected to reach people through live, public, events, but this was not feasible in Ghana since Ghanaian girls’ mobility was low.

Evaluators used data from the baseline household survey to conduct segmentation analysis - which groups individuals into a small number of homogenous segments – to aid campaign message development. This helped the implementer develop an understanding of sub-groups within the population who could be targeted with messages that may be particularly appealing to them. The implementer also conducted focus group discussions (FGDs) to understand how exposure to certain project activities, such as the movie or brand activations, were received. Continuous listening to adolescents’ response to project activities through quantitative (and some qualitative) tools helped ensure that implementers understood the social and cultural context of adolescent girls’ behavior. In Botswana, adolescent girls spent more time out of the house with their friends than in Ghana where girls were more influenced by parents who exercised greater control over their movement. This made reaching adolescent girls through social media challenging in Ghana and shifted the project’s focus towards school-based activities. There was also a more direct attempt to keep parents in Ghana informed and to secure their support for SKY.

**Figure 2.**
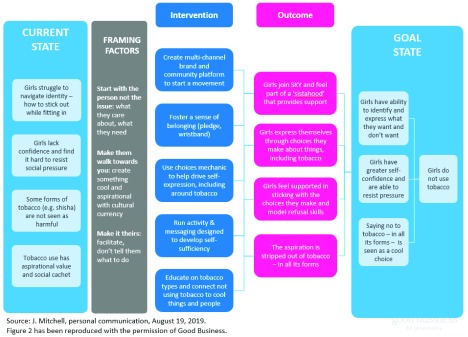
Evolved theory of change for SKY girls tobacco control intervention.

The Ghanaian experience helped intervention designers to modify their initial ToC. Figure 2 shows the modified ToC of the SKY Girls tobacco control intervention. The modified ToC included a greater emphasis on the risk associated with certain types of tobacco, notably *shisha*. The outcome measures were tightened and rationalized in the new version of the ToC. Finally, the modified ToC took the desired behavior, non-use of tobacco by teenage girls, explicitly into account.

## Discussion

The feedback loop established in Ghana – a combination of the use of phone survey data for programmatic purposes and feedback gathered through the implementers’ regular interaction with the audience through in-person and online interactions - enabled GB to implement a different intervention than what they would have implemented in the absence of insights about the consumer.

A key feature of the feedback loop was the close involvement of the donor at all stages of project design, implementation and evaluation. Decisions could be taken quickly since the donor understood the types of issues that came up on the implementation and the evaluation side. This allowed implementers to shift to new channels and strategies that were more suitable to the Ghanaian context. The creation of free full-length movies is one such adaptation; the movies spoke directly to the Ghanaian teen audience, access and screening were designed around community needs, and the channel worked well with the evaluation design – distribution could be closely managed to minimize contamination of the control, non-project areas. Evaluators appreciated that the donor team did not micromanage the project, enabling them to have flexibility to make changes to the surveys when needed and possible. They felt that the inclusion of an evaluation expert on the donor team made it easier for them to provide the rationale for their analyses and survey design choices to implementers and for the intent-to-treat approach used for analyses of impact using the baseline and endline household surveys.

### What didn’t work as well

Feedback loops worked only partially at the outset of the project: while the evaluators did discuss the quasi-experimental design of the evaluation and its reliance on population-based representative surveys during the December 2016 face-to-face meeting, in-depth input on the evaluation design could not be obtained from implementers because of their lack of familiarity with a quasi-experimental design and the type of data it generates.

Low exposure to project activities in the first telephone survey (conducted about 2–3 months into the project) did not ring true to the implementers who were used to relying on project-generated outputs (such as numbers of people exposed to project activities or discussions on the Facebook page) that are not population-based. Implementers also had concerns about the reliability of the phone survey data, since a higher proportion of girls reported exposure to SKY on television than they expected. Evaluators felt that had they been able to work more closely with the implementers in discussing the evaluation design and the types of results it could generate at the project’s outset, the study design could have been adjusted to provide data that would have been even more useful to implementers. Additional face-to-face meetings between evaluators and implementers at the project’s outset may also have resulted in more insights with practical implications for the intervention.

Implementers felt that had population-based survey findings been available to them earlier - there was a 2-month gap between data collection for the baseline household survey and availability of the first set of results showing that radio and Facebook exposure was low among teens - they would have been able to make changes to intervention design sooner. Implementers did start making changes to the intervention design within 3 months of the launch of the campaign.

Implementers appreciated the evaluators’ explaining the study design to them at the start of the project. Implementers also felt that the initial face-to-face meeting with the evaluators made a big difference to their working relationship. It deepened the understanding of each other’s work and increased the willingness to be flexible in the design of both the survey and the intervention in support of the end goal and made subsequent communication easier. For example, implementers felt that they could request the evaluators to add specific questions that were important from an implementation perspective to phone surveys.

Implementers found that quantitative surveys were not ideal in helping them gain an understanding of adolescent girls’ behavior. Their experience of focus group discussions (FGDs) with very young adolescents in Botswana had showed them that girls who initially reported being non-smokers would then reveal that they smoked once they became more comfortable with the moderator. Implementers felt that greater use of qualitative methods in the evaluation would have been helpful. They were concerned about the length of the quantitative household survey questionnaire and the duration of time spent in the interview by such a young population. The survey questionnaire took an average of 40 minutes in Ghana. Their experience with a similar age group in Botswana had shown that very young adolescents started losing interest in responding to a questionnaire after about 10 minutes. Indeed, the low reported rates of actual smoking at baseline and endline surveys show the limitations of a quantitative survey implemented in a home setting in being able to capture a hidden behavior such as teen smoking.

In spite of the regular communication between implementers and researchers, evaluators did not learn about one of the project’s new activities that was piloted during the last 4 months of the campaign –- community activation trucks to reach teens in Ghana – until near the end of the implementation period. Evaluators were, however, still able to include a question measuring exposure to this activity in the endline household survey. Had evaluators known of this activity earlier, they would have been able to measure exposure to it through the mobile phone surveys as well.

By asking a few open-ended questions in the quantitative surveys, evaluators were able to determine that the indirect approach taken by implementers towards convincing girls not to smoke by focusing on the negative physical aspects of smoking was working: teens gave avoidance of getting yellow teeth and bad breath as their main reasons for not smoking. The emotional appeal to teen girls of maintaining their attractiveness appears to have worked.

Evaluators felt that implementers found the evaluation design to be very challenging. The evaluation focused on measuring population-level coverage. However, this did not provide insightful information for implementers during the initial phase of implementation - since exposure to project activities was very low initially. In other words, although the surveys employed relatively large sample sizes (over 2,000 for telephone surveys), low exposure to the intervention at project beginning meant that little analysis could be done around message comprehension (since there were not enough girls exposed to the campaign in the sample survey).

Evaluators felt that they could have been more helpful if they had been more successful in explaining what could and could not be obtained from the evaluation design. They felt that a more flexible evaluation design would have been more suitable than the fixed, quasi-experimental design that they had chosen. They did, however, make the best use of all available data by conducting additional analyses of data from the project database, additional analyses of baseline household survey data and data from the phone surveys. For example, evaluators substituted one of the mobile phone surveys which was based on a sample of respondents from the household survey for a mobile phone survey which was based on a sample of respondents from the project activities database. The latter database collected data on teens exposed to project activities.

## Data availability

No data are associated with this article.
