# Supplementary material for: An illustration of how responsive feedback in a social marketing tobacco control intervention in Ghana enabled managers to make decisions that increased intervention effectiveness
Source: Gates Open Res. 2019 Sep 9;3:1534. [Version 1] doi: 10.12688/gatesopenres.13062.1 (PMC6904888; doi:10.12688/gatesopenres.13062.1)
Supplement: Peer review history

# Peer review: Reviewer response for version 1

**DOI:** 10.21956/gatesopenres.14193.r28138

**Published:** 2019-12-10

**Authors:** Kenneth Sherr

**Affiliations:** 1 Department of Global Health, University of Washington, Seattle, WA, USA

This paper describes the process through which a social marketing approach developed in Botswana was transferred to Ghana, with refinements in strategy informed from an iterative relationship and information sharing between program implementers and evaluators. The paper is well written and informative, and is an interesting case study in that it captures a unique case where a relationship that is often kept at arm’s length (implementers, evaluators and donors) is instead united to lead to a more evidence-based adaption. 

- It would be helpful to introduce how the data that informed this case study were collected and analyzed. Does it represent only the perspective of the authors, or was there a more systematic approach to data collection and interpretation?
- There is a bit of a contradiction in the description of the tenor of the relationship between the implementing team and evaluating teams, which merits clarification and nuance. On page seven, there were concerns expressed from the implementing team that community survey results were late to be shared, and therefore not used to alter the program strategy. But the next paragraph notes that the implementers had a constructive relationship with the evaluative team. These two statements are at odds with each other.
- I think the discussion could be bolstered with a deeper exploration of the applicability of the case study lessons to other situations or programmatic/geographic areas. This case study really focused on how channels of communication or messages were altered or changed in emphasis, which makes sense given that it was a social marketing intervention. Would a similarly iterative approach work for non-behavioral intervention refinement? How could it be useful for supply side/health system improvement strategies? 

I confirm that I have read this submission and believe that I have an appropriate level of expertise to confirm that it is of an acceptable scientific standard.

# Peer review: Reviewer response for version 1

**DOI:** 10.21956/gatesopenres.14193.r28137

**Published:** 2019-11-11

**Authors:** Saugato Datta

**Affiliations:** 1 Ideas42, New York, NY, USA

The article describes a process through which the authors, who were part of a team funding an evaluation of a behavior-change communications campaign targeting adolescent girls' (potential) tobacco use in Ghana, which was in turn based on a previously tested, successful such intervention in Botswana. The key contribution of this article is to describe how data/insights from a feedback loop set up for various parties to the intervention/evaluation was used to adapt the content and mode of delivery of the intervention being evaluated in a way that is likely to have improved its suitability to the context and its effectiveness. 

 The article is well written and clearly walks the reader through the process by which the team utilized the data and insights emerging as part of the implementation of the intervention to identify ways in which needed to be adapted, and to facilitate these changes. It also identifies ways in which the feedback mechanisms set up and utilized did not work as well as intended, and offers suggestions for learning from these insights.

 Overall, I found the article to be a useful and thought-provoking read. I will summarize below some thoughts on specific and broader ways in which I think it could be further improved.

 Specific suggestion:

1. The authors describe the previous intervention in Botswana as "changing knowledge, attitudes, and perceptions around tobacco use", and refer to measurements of effects of the campaign on "indicators such as the number of girls who spontaneously listed smoking as something they were opposed to and the number of girls who thought smoking made them more attractive". However, they then refer to the ability to track behavior of teens exposed to the intervention. It seemed at least to this reader that the article would benefit from making a clearer distinction between knowledge or attitudes or perceptions (which the intervention targets) and tobacco-related behaviors, e.g., the (non)uptake, (dis)continuation etc of tobacco use, (which it does not measure, as far as I could tell). Recognizing the inherent difficulty of capturing not only measures of such behaviors but also the extreme difficulty of establishing a counterfactual in a case such as this (where the appropriate measure of behavior change would seek to capture behavior that did not take place but, absent the intervention, would have), I do not think the focus on attitudes, beliefs, etc is in any way a problem, but would merely suggest that the distinction between these and behavior be carefully acknowledged and not elided. 
2. The authors might want to refer to, and possibly cite, recent research which also takes seriously the need for field-based inputs into the development and refinement of behavior change interventions. An example is Buttenheim *et al*. (2019) ^1^, which takes a similarly thorough approach to intervention design and refinement. It would be interesting to see the authors situate this research within the broader body of literature that engages carefully with the need to ensure that interventions are adapted to the context they are being implemented in.

Beyond these specifics, the article also raised a broader question for me, which it would be interesting to have the authors reflect upon in the article should it be revised further. This is about the order in which decisions about what to implement to tackle a problem in a given context ought to be taken. While the article does a great job of showing us how data and feedback can make an intervention more robust, it also raised questions for me that an intervention developed in Botswana was sought to be evaluated in a new context, i.e. Ghana, without initial investigative work around critical pieces of the new context, such as teenagers' preferred modes of social or conventional media engagement (e.g., TV, radio, etc). While the iterative feedback loops established for the project clearly helped the team adapt to critical differences between the previous and proposed context, the article left me with a niggling set of queries about why an evaluation was proposed/greenlighted without this sort of investigation of the context having been carried out as a preliminary step. It would be interesting for the authors to address this question head-on, because it would appear to speak to a key set of questions around the ways in which the questions the field asks 'Does this intervention, which worked in Country X, work in Country Y"? may be in need of revision (perhaps, for example, we should instead ask ''Do the conditions that made this Intervention, which worked in Country X, exist in the same way in Country Y? And if not, how should we revisit the motivating question and intervention design").

I confirm that I have read this submission and believe that I have an appropriate level of expertise to confirm that it is of an acceptable scientific standard.

## References

[1] : A behavioral design approach to improving a Chagas disease vector control campaign in Peru. *BMC Public Health*.2019;19(1) : 10.1186/s12889-019-7525-3 1272 10.1186/s12889-019-7525-3 31533762PMC6751594
